# Book Review: Language Education and Emotions: Research Into Emotions and Language Learners, Language Teachers and Educational Processes

**DOI:** 10.3389/fpsyg.2021.732706

**Published:** 2021-08-30

**Authors:** Zaibo Long, Jinfen Xu

**Affiliations:** ^1^School of Foreign Languages, Wuhan University of Science and Technology, Wuhan, China; ^2^School of Foreign Languages, Huazhong University of Science and Technology, Wuhan, China

**Keywords:** language education, teacher emotions, educational processes, positive emotions, learner emotions

Recent years have witnessed an explosion of interest in how emotions are of vital importance in language education. This trend motivates researchers to investigate “what goes on inside and between the people in the classroom”(Stevick, 1980: 4) that influences the success of language learning. The book under review follows this trend and provides illuminating insights into the emotional aspects of language education.

The edited volume consists of an introductory chapter (Chapter 1), another 10 chapters structured into three parts, and a concluding one.

Jane Arnold, in Chapter 1, sketches out an informative overview of affect in language learning, arguing for the inseparability of affect and cognition. According to Arnold, language learning affect (In the volume, the terms *affect* and *emotion* are used interchangeably) involves two dimensions, the *inside* and the *between*. While the *inside* dimension refers to such individual factors as motivation, confidence and self-esteem, and anxiety, the *between* dimension concerns the interpersonal and relational factors. Both dimensions are claimed to be crucial for successful language learning.

The first part of the volume (Chapters 2–5) deals with learner emotions. Chapter 2, by Jakub Bielak and Anna Mystkowska-Wiertelak, presents a review on studies of emotion regulation strategies. The authors summarize a systematic list of emotion regulation strategies both in general education and in language learning. However, they lament the limited number of studies that specifically investigate the use and effectiveness of emotion regulation strategies in the unique processes of language learning. In an attempt to examine the effectiveness of an individualized approach to self-regulated learning strategy instruction, Shenglan Zhang in Chapter 3 documents an empirical study and shows that the instruction was helpful not only for students to find and apply effective self-regulated learning strategies, but also for them to build positive emotions. Chapter 4, by Blanca Cristòfol Garcia and Christine Appel, reports a study on the factors influencing learner anxiety in the context of synchronous computer-mediated interaction. The study identified more anxiety-reducing factors than anxiety-triggering ones in this context. Karin Vilar Sánchez in Chapter 5 demonstrates how the feeling of recognition and appreciation of Spanish migrants in Germany helped to reduce the possibility of being negatively affected by German traits of relative directness and reservedness.

The second part of the volume (Chapters 6–8) concerns teacher emotions. Marie-Claire Lemarchand-Chauvin, in Chapter 6, explored which emotions novice English as a foreign language teachers experienced at work, and how self-confrontation interviews could affect their valence of emotions. The study revealed that they mainly experienced negative valence emotions, and that the self-confrontation interviews could be a powerful tool for them to reflect on their practices and to shift their initial emotions. Soufiane El Ouastani in Chapter 7 delved into non-native foreign language teachers' experiences of anxiety. He found that the majority of participants experienced average levels of anxiety, and the causes of their anxiety included their irrational analysis of their target language abilities, their cultural and linguistic background, their attachment to the target language etc. Majid Elahi Shirvan and Tahereh Taherian, in Chapter 8, investigated how a teachers' self-disclosure in classroom interaction contributed to students' foreign language enjoyment. The study highlights the importance of teachers invoking different identities embedded in their self-disclosure in creating a supportive climate for learners to freely use the target language for communication.

The third part (Chapters 9–11) focuses on emotions in educational process. Andrea Bicsar, in Chapter 9, examined how social distance and dominance as basic contextual parameters influenced Austrian classroom English learners' lexical choices in talking about emotions in English. The study underscores the necessity of pragmalinguistic as well as sociopragmatic knowledge for foreign language learners to successfully verbalize emotions. Marie Ploquin, in Chapter 10, explored the effects of two English pronunciation learning strategies (self-recording and listening vs. simply repeating without self-recording) on learner motivation and self-confidence. It was found that both strategies evoked positive emotions, whereas the former was applauded as more advantageous by learners. Arguing for an equilibrium of emotional and rational learning in content and language integrated learning (CLIL) in the social sciences so as to promote global justice and solidarity, Subin Nijhawan, in Chapter 11, probed how a judicious and principled integration of learners' first language into CLIL classes contributed to this equilibrium.

The volume concludes with Jean-Marc Dewaele's review of the status quo of the emotion research in language education. The review shows how emotions are contagious, underlining the importance of emotional regulation for both teachers and learners. At the end of the review, Dewaele advocates the necessity to delve into the bidirectional emotion interaction patterns between teachers and students in various contexts.

The volume contributes to a holistic and ecological view of how emotions play a crucial role in language education. First and foremost, it not only covers learners and teachers' emotions separately, but also emphasizes that the two types of emotions interact with each other, and that emotions are malleable, indicating possibilities to cultivate an agreeable atmosphere for developing learner emotions that are conducive to learning. Secondly, the volume redresses the bias in the past few decades that isolates emotion and cognition (Xu and Long, 2020), insisting that “without emotion there will not be elements such as curiosity, attention and memory which are necessary for the learning process” (p. 7). This acknowledgment of the integrity of cognition and emotion renders new insights into the nature of language teaching and learning, which might raise readers' awareness of the long-neglected emotional elements in learning and motivate teachers to take proper actions to address them. Lastly, the volume avoids a binary division of negative and positive emotions, but instead revolves around all types of emotions in diverse pedagogical contexts. This stance enables readers to get a full picture of the role of emotions in language education.

Despite the above-mentioned merits, what one might regret is the absence of chapters introducing classroom interventions designed to enhance learners' positive emotions or reduce their negative ones. Such chapters might add to the value of the book.

Nonetheless, the book is worth recommending to language teachers, researchers, teacher educators and postgraduates who are interested in understanding and exploring emotional aspects of language education.

## Author Contributions

ZL wrote the initial draft of the book review. JX provided several rounds of critical feedback to the drafts in the process of writing. The final draft is the result of the ZL and JX collective effort. All authors contributed to the article and approved the submitted version.

## Conflict of Interest

The authors declare that the research was conducted in the absence of any commercial or financial relationships that could be construed as a potential conflict of interest.

## Publisher's Note

All claims expressed in this article are solely those of the authors and do not necessarily represent those of their affiliated organizations, or those of the publisher, the editors and the reviewers. Any product that may be evaluated in this article, or claim that may be made by its manufacturer, is not guaranteed or endorsed by the publisher.
